# Pharmaceuticals in treated wastewater induce a stress response in tomato plants

**DOI:** 10.1038/s41598-020-58776-z

**Published:** 2020-02-05

**Authors:** Rena Gorovits, Iris Sobol, Kazuhito Akama, Benny Chefetz, Henryk Czosnek

**Affiliations:** 1 Institute of Plant Sciences and Genetics in Agriculture, Robert H. Smith Faculty of Agriculture, Food and Environment, The Hebrew University of Jerusalem, Rehovot, 76100 Israel; 2 Department of Biological Science, Shimane University, Matsue, Shimane 690-8504 Japan; 3 Institute of Soil and Water sciences, Robert H. Smith Faculty of Agriculture, Food and Environment, The Hebrew University of Jerusalem, Rehovot, 76100 Israel

**Keywords:** Plant sciences, Abiotic

## Abstract

Pharmaceuticals remain in treated wastewater used to irrigate agricultural crops. Their effect on terrestrial plants is practically unknown. Here we tested whether these compounds can be considered as plant stress inducers. Several features characterize the general stress response in plants: production of reactive oxygen species acting as stress-response signals, MAPKs signaling cascade inducing expression of defense genes, heat shock proteins preventing protein denaturation and degradation, and amino acids playing signaling roles and involved in osmoregulation. Tomato seedlings bathing in a cocktail of pharmaceuticals (Carbamazepine, Valporic acid, Phenytoin, Diazepam, Lamotrigine) or in Carbamazepine alone, at different concentrations and during different time-periods, were used to study the patterns of stress-related markers. The accumulation of the stress-related biomarkers in leaf and root tissues pointed to a cumulative stress response, mobilizing the cell protection machinery to avoid metabolic modifications and to restore homeostasis. The described approach is suitable for the investigation of stress response of different crop plants to various contaminants present in treated wastewater.

## Introduction

Expanding populations aggravates the demand for freshwater for human consumption, agriculture and industry. While the sources of freshwater are limited and often polluted, reclaimed wastewater offers an additional resource that could be used to irrigate crops in agricultural systems. However, only 1% of the water used for irrigation worldwide consists of treated wastewater. Several countries use reclaimed wastewater for crop irrigation. For example, Israel uses 85% of the treated wastewater for irrigation, over half of the total irrigation volume^1^. However, the current water purification devices are unable to eliminate some pharmaceuticals used by humans for personal care and medicine, released in the sewage. With the growing use of treated wastewater for irrigation, increasing amounts of these products find their way in the agroecosystem^2^. The fate of these pollutants on soils and on plants^3^, and the potential risks for humans ingesting the plant edible parts (from infants to pregnant women and seniors) are under scrutiny^4–6^. Therefore, the use of treated wastewater in the EU could be regulated in the near future^7^.

Psychoactive drugs are a major class of pharmaceuticals still found after wastewater treatment^8,9^. They originate from excretion by patients, discarded medications or pharmaceutical industry effluents. Among them, Carbamazepine (CBZ) is one of the most abundant. This drug is used to treat epilepsy and trigeminal neuralgia. Low removal efficiency and minimal degradability of CBZ and its metabolites have been reported^10,11^. CBZ is detected in influent and effluent wastewater in municipal wastewater-treatment plants in many countries^10,12–14^. For example, in the city of Peterbourg, ON, Canada, the concentration of CBZ and its derivatives was about 460 ng/l in the untreated wastewater and 440 ng/l after treatment^10^. CBZ is highly persistent in soils where its biodegradation is limited^15,16^, and it accumulates in a variety of crops^17–20^. CBZ is readily detected in treated wastewater used for irrigation. In recent experiments conducted in Israel^21^ the concentration of CBZ and derivatives in treated wastewater was 1.3 to 2.2 μg/l before application to soil; it was 100 to 150 ng/g (dry weight) in leaves of tomato grown on various soils in lysimeters and irrigated with these treated wastewaters for about 100 days.

Other pharmaceutical drugs, often detected in wastewater, include Lamotrigine (LTG), Valproic acid (VAL), Phenytoin (PHY), and Diazepam (DZP)^22^. LTG is an antiepileptic drug, sometimes co-administered with CBZ. VAL is used to treat epilepsy and bipolar disorder and to prevent migraine headaches. PHY is an anticonvulsant helping control seizures. DZP, also known as valium, is used to treat anxiety among other conditions and has a calming effect on spasms and seizures.

In a recent review^1^ the current knowledge and the knowledge gaps pertaining to reclaimed wastewater irrigation practices were summarized. It was mentioned that information on effect of the compounds remaining in treated wastewater on terrestrial plants was limited. The present understanding on the effects of the compounds on plants was classified as “low”. The present study aimed at filling some of these gaps.

It appears that many compounds remaining in treated wastewater induce detoxification processes in plants, which include enhanced activities of detoxification enzymes such as superoxide dismutase, peroxidase and glutathione-S-transferase^23,24^. Lately, it was asked whether these pollutants could be considered as plant stressors^25^. To cope with various stresses, plants have developed a comprehensive quality control system aimed at maintaining protein homeostasis and improving plant survival. In plants, the general stress response includes the overproduction of reactive oxygen species (ROS), which activate the mitogen-activated protein kinase (MAPK) signaling cascade, the upregulation of chaperone/heat shock proteins (HSPs) genes, the activation of elements of the γ-aminobutyric acid (GABA) shunt (including the glutamate decarboxylases GAD1 and GAD2), and the accumulation of amino acids and sugars involved in maintaining the cells osmotic balance^26–28^.

In this report, we considered pharmaceutical compounds present in treated wastewater as stress inducers. We have watered tomato seedlings with pharmaceuticals found in treated wastewater used for irrigation and we have confronted our results with a number of criteria characterizing a general plant stress response. To improve our understanding of how pharmaceuticals interact with biochemical pathways of plants, we studied the patterns of stress protein biomarkers, such as HSPs and MAPKs, GABA-shunt components including key enzyme of GABA biosynthesis, glutamate decarboxylase (GAD1 and 2), as well as several of other stress-induced amino acids. Changes in these molecular stress markers were conspicuous in leaf and root of tomato seedlings treated with a mix of pharmaceutical compounds (CBZ, LTG, VAL, PHY, DZP) and with CBZ alone. Therefore, we conclude that pharmaceuticals present in treated wastewater induce a typical stress response in tomato plants.

## Results

### Incubation of tomato seedlings with a cocktail of pharmaceutical compounds induces short and long-term changes in stress response markers

#### Choice of pharmaceutical concentration

Pharmaceuticals and personal care products concentrations range from ng to μg/l in treated wastewater^10,21^. Roots of tomato seedlings were bathing in a mix of CBZ, LTG, VAL, PHY, DZP; each compound in the mix was at a concentration of 0, 10, 100 and 1000 ppb (1 ppb is 1 μg/l). The concentration of pollutants was well above that found in treated wastewater in order to enhance the stress response and to identify the processes involved. In a comparable experiment, published in 2018^24^, cucumber seedlings in a hydroponic setting were exposed to a mixture of 17 pollutants (including CBZ and DZP) at concentrations of 0, 0.5, 5 and 50 μg/l. At 0.5 and 5 μg/l, ROS levels in roots and shoots increased several folds. At 5 and 50 μg/l, an increase in the activity of antioxidant enzymes such as peroxidase and glutathione-S-transferase was observed.

**Figure 1:**
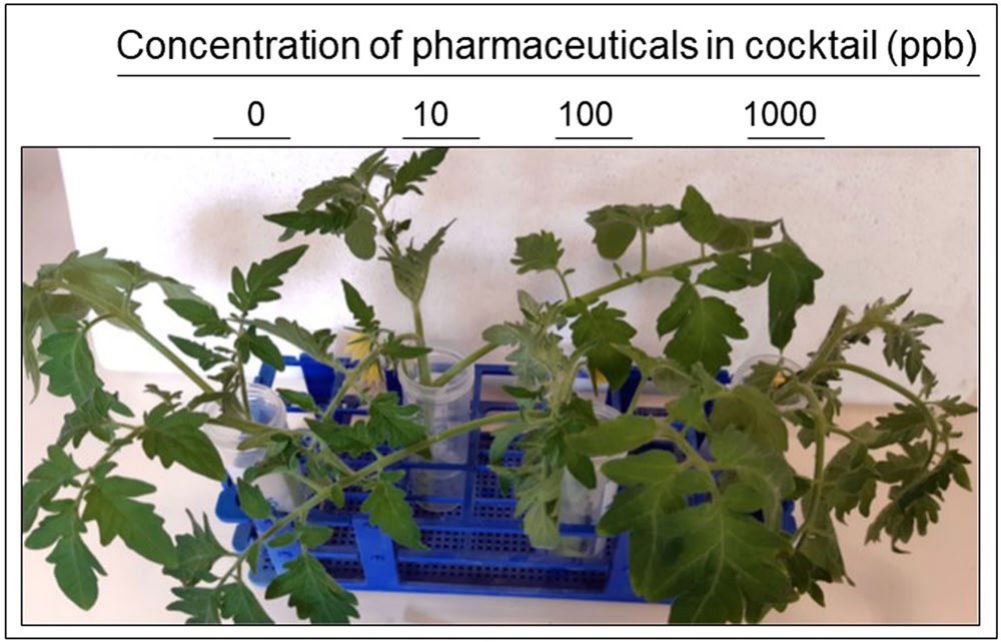
Appearance of tomato seedlings with roots bathing in a pharmaceutical cocktail. Roots bathing for one week in a cocktail of Carbamazepine, Valporic acid, Phenytoin, Diazepam, and Lamotrigine, at concentration of 0 (tap water), 10, 100 and 1000 ppb. Note the plant decaying with the 1000 ppb treatment.

After five days, the tomato seedlings treated with 1000 ppb have collapsed. In contrast, the plants treated with 10 and 100 ppb performed as well as the control plants bathing in water (Fig. 1). Therefore, 10 and 100 ppb were used for further analyses. Each experiment contained control plants incubated in the exactly same conditions (in 50 ml falcons with tap water refreshed every 2 days). The leaf and root samples of control plants were used to level off any stress caused by the experimental conditions.

#### Induction of stress-associated proteins with 100 ppb pharmaceutical cocktail

**Figure 2:**
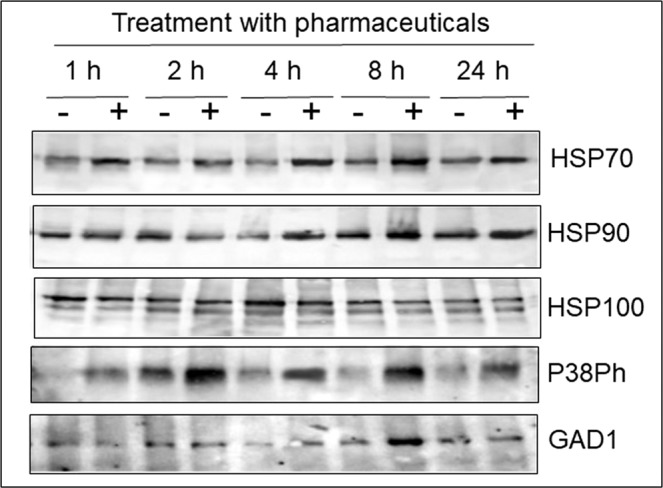
Western blot analyses of tomato leaf stress-related protein profiles upon treatment with the pharmaceutical cocktail at 100 ppb. Roots of tomato seedlings were bathing in tap water (−) and in a cocktail of Carbamazepine, Valporic acid, Phenytoin, Diazepam, and Lamotrigine, at concentration of 100 ppb (+). Total proteins were extracted from leaves harvested after 1, 2, 4, 8 and 24 h. Each set of samples was subjected to electrophoresis in five identical gels. Each gel was blotted and reacted with a different stress-specific protein antibody raised against chaperones (HSP70, HSP90, HSP100), the member of the MAPK family P38, and of the GABA-shunt enzyme glutamate decarboxylase 1 (GAD1). The photographs show the relevant region of the gel.

Total proteins were extracted from tomato leaves harvested before, and after short (1, 2, 4, 8 and 24 h) treatment with the pharmaceutical cocktail. The proteins of interest were immunodetected in western blots using specific antibodies (Fig. 2). The amounts of two main chaperones, HSP70 and HSP90, but not HSP100, increased in the leaf during the treatment. From the MAPKs family, only P38Ph accumulated upon the treatment, not ERKPh and JNKPh (not shown). The amount of GABA-shunt enzyme glutamate decarboxylase 1 (GAD1) slightly increased after 8 h of cocktail treatment.

**Figure 3:**
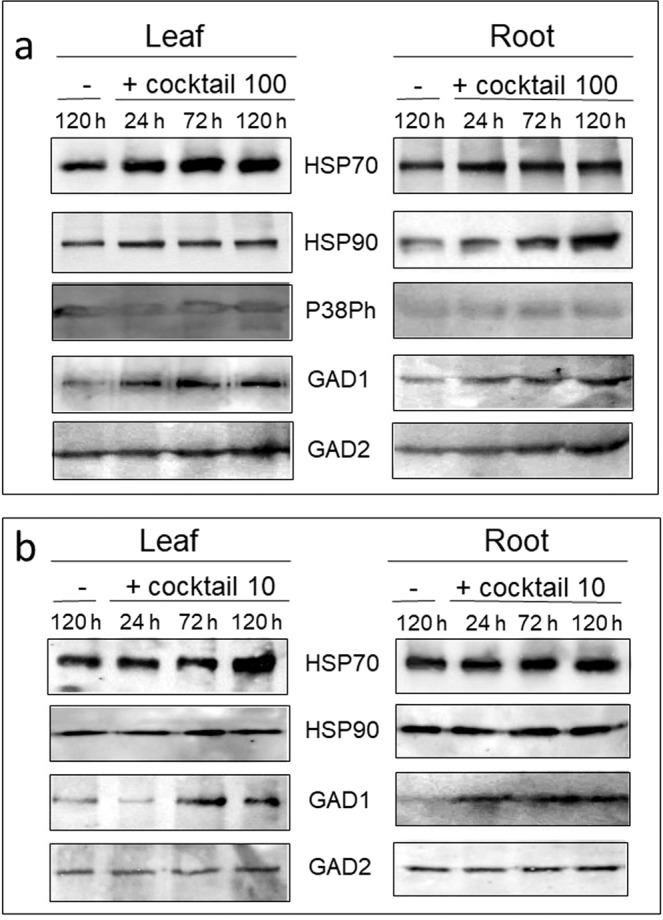
Western blot analyses of tomato leaf and roots stress-related protein profiles upon treatment with the pharmaceutical cocktail at 100 (**a**) and 10 (**b**) ppb. Roots of tomato seedlings were bathing in tap water (−) and in the cocktail of Carbamazepine, Valporic acid, Phenytoin, Diazepam, and Lamotrigine, at concentration of 100 (**a**) and 10 ppb (**b**). Total proteins were extracted from leaves and roots harvested after 24, 72 and 120 h (also served as control of roots bathing in tap water (−). Each set of samples was subjected to electrophoresis in five or four identical gels. Each gel was blotted and reacted with a different stress-related protein antibody raised against chaperones (HSP70, HSP90), the member of the MAPK family phosphorylated P38 (P38Ph), and of the GABA-shunt enzymes glutamate decarboxylases 1 and 2 (GAD1 and GAD2). The photographs show the relevant region of the gel.

The long-term response of the tomato seedlings to the same pharmaceutical cocktail (100 ppb of each drug) was analyzed following treatment during 24, 72 and 120 h (when the plant starts to decay), not only in the leaf, but also in the roots (Fig. 3). Proteins extracted from plants bathing in water for 120 h were used as controls. HSP70 accumulated in the leaf, and to a lesser extent, in the roots (Fig. 3a). In contrast, HSP90 amounts increased in root, less in leaf. Two GABA-shunt enzymes, GAD1 and GAD2, accumulated in greater amounts in roots than in leaf (Fig. 3a). In contrast to P38Ph induction during the first 24 h of treatment (Fig. 2), the amounts of P38Ph did not change during the long-term treatment (Fig. 3a). It is possible that after 72 h of treatment, the changes in phosphorylation are erratic and are not always identified. To examine this hypothesis, P38Ph was immuno-tested shortly after 72 h of cocktail treatment, at 73, 74, 76 and 80 h. In two independent experiments (I and II), it can be see (Supplemental Fig. 1a) that in one case (at 80 h), the amount of P38Ph did increase.

#### Induction of stress-associated proteins with 10 ppb pharmaceutical cocktail

To bring our experiments closer to conditions experienced by tomato plants growing in fields irrigated with treated wastewater (the concentration of CBZ and derivatives in treated wastewater in Israel is in the μg/l range^21^, the concentration of the cocktail of the five pharmaceuticals was decreased to 10 ppb (10 μg/l), instead of 100 ppb. The effect of the cocktail for up to 120 h on stress-response proteins was appraised. The stress response to 10 ppb cocktail was less prominent than to the 100 ppb cocktail (Fig. 3b). The amount of HSP70 increased only at 120 h in leaf, but not in root. HSP90 and GAD2 no longer showed any change, while the amounts of GAD1 increased in both leaf and root. The results showing the accumulation of protein stress markers, HSPs and GADs, indicate that pharmaceutical compounds found in treated wastewater cause a permanent stress response in tomato leaf and root.

#### Amino acids patterns

**Figure 4:**
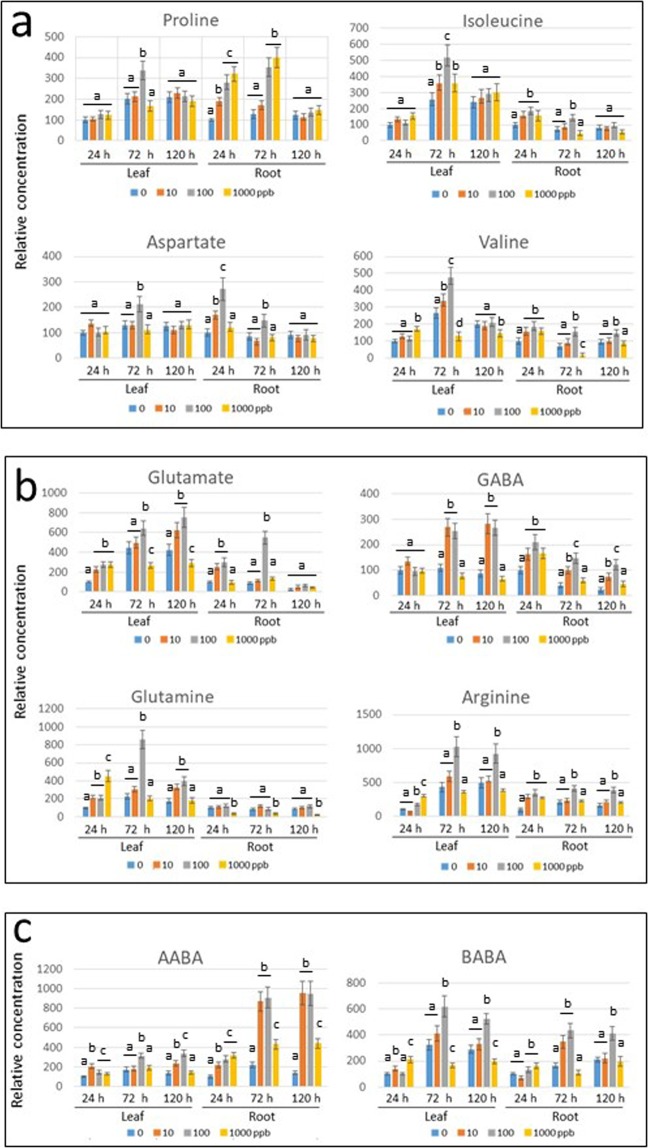
Amino acid profile of tomato leaf and roots of roots bathing in pharmaceutical cocktail at 0 (tap water), 10, 100 and 1000 ppb. The leaves and roots were sampled after 24, 72 and 120 h. The concentration of amino acids in leaf and root of plants incubated in water was considered as 100; the concentrations of amino acids were calculated relative to this value. **(a)** Osmo-protectants, which include Pro, Ile, Asp, Val. **(b)** Glutamate family of amino acids, which includes Glu, Gln, GABA and Arg, common osmolytes accumulated by plant cells in response to stress. **(c)** Non-protein amino acids α- and β-aminobutaric acids (AABA and BABA, respectively) involved in pathogen resistance. Three independent experiments were performed. The amounts at every time-point/pollutant concentration were measured three times. Bars represent the standard errors, and different lowercase letters (a–d) above the bars denote significant differences (p < 0.05).

In order to understand the metabolism of amino acids in response to pharmaceutical treatments, the amounts of amino acids in leaf and root were measured 24, 72 and 120 h after the beginning of plant irrigation with 0, 10, 100 and 1000 ppb concentrations of pharmaceutical cocktail (Fig. 4). Even though tomato seedlings decayed after five days incubation in cocktail of 1000 ppb (Fig. 1), we analyzed the patterns of selected amino acids. The concentration of amino acids in leaf and root of plants incubated in water was considered as 100; the concentrations of amino acids from plants irrigated with the pharmaceutical cocktails were calculated relative to this value. The comparison was between the different concentrations of pharmaceuticals within each time point, in leaf and root.

Pro is considered as a marker of osmotic stress response, together with several other amino acids known to be plant osmo-protectants such as Val, Ile and Asp (Fig. 4a). In leaves, the concentration of Pro did not change significantly, while in the roots, there was a concomitant intensification with the increase of the amount of pharmaceuticals in the cocktail and with the duration of exposure. At 120 h of treatment, the plants had ceased to respond to the stress (likely because they started to decay, Fig. 1).

Ile concentrations increased in the leaves with plant development, but not in the roots. Upon treatment with the pharmaceutical cocktail, increased concentrations of Ile were measured in the leaves after 72 h, but not in the roots. Similar to Pro, the plants had ceased to respond to the stress after 120 h treatment. Val presented a response similar to that shown for Ile. The concentrations of Asp increased in leaf and root of plants subjected to amounts of pharmaceuticals of 100 ppb for 24 or 72 h.

These results showed that upon pharmaceutical exposure, the concentrations of amino acids involved in osmo-protection increase up to 72 h of treatment. The effect is more noticeable when pharmaceutical concentrations are 100 ppb, in leaf and root as for Pro and Asp, in leaf only as for Ile and Val.

Pro belongs to the glutamate family of amino acids, which includes Glu itself, Gln, GABA and Arg. The concentrations of Glu increased in leaves of plants exposed to cocktails of pharmaceuticals at concentrations of 10 and 100 ppb, while in roots the changes were less pronounced (Fig. 4b). The concentrations of Gln and Arg were enhanced in leaf, those of arginine slightly increased in root. GABA was up-regulated by 10 and 100 ppb cocktail in leaf and in roots.

Two non-protein amino acids α- and β-aminobutaric acids (AABA and BABA, respectively) are known to protect plants against different pathogens. In particular, BABA was shown to enhance Arabidopsis resistance to microbial and fungal pathogens, as well as to abiotic stresses (35 and references therein). AABA concentrations greatly increased in root, to a much lesser extent in leaf, at all pharmaceutical concentrations. The induction of BABA was conspicuous in leaf and in roots especially after 72 h of treatment (Fig. 4c).

Several additional amino acids responded to the pharmaceutical treatment (Supplemental Fig. 2A). Lys and Leu accumulated to greater concentrations in leaf (not in roots) upon pharmaceutical treatment than in untreated plants. The effect on Thr and Cys was marginal, even though Lys and Thr are known to be stress-induced metabolites.

### Changes in the patterns of stress response markers during short- and long-term treatments with different concentrations of Carbamazepine

#### Protein patterns

Among the compounds constituting the pharmaceutical cocktail, we choose to study the effect of CBZ in more details. since CBZ is a major pollutant readily found in treated wastewater^19^. Tomato seedlings were incubated in different concentrations of CBZ (10, 200, 1000 nM) during different times (from one hour to five days).

**Figure 5:**
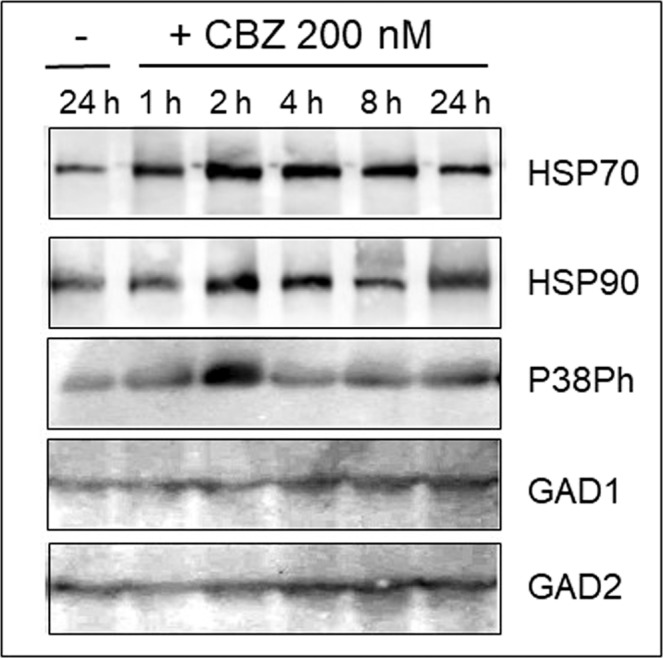
Western blot analyses of tomato leaf stress-related protein profiles upon treatment with 200 nM Carbamazepine (CBZ) for up to 24 h. Roots of tomato seedlings were bathing in tap water (−) and in CBZ (+). Total proteins were extracted from leaves harvested after 1, 2, 4, 8 and 24 h (roots bathing in tap water for 24 h served as control). Each set of samples was subjected to electrophoresis in five identical gels. Each gel was blotted and reacted with a different stress-related protein antibody raised against chaperones (HSP70, HSP90), the member of the MAPK family phosphorylated P38 (P38Ph), and of the GABA-shunt enzymes glutamate decarboxylases 1 and 2 (GAD1 and GAD2). The photographs show the relevant region of the gel.

Already after 2 h incubation of seedlings in 200 nM CBZ, the chaperones HSP70 and HSP90 were induced in leaf tissues (Fig. 5). A similar kinetics of induction was detected in plants treated with 1000 nM CBZ (not shown). HSP70, but not HSP90, was induced by 10 nM CBZ (Supplemental Fig. 1b). P38Ph MAPKs was induced only after 2 h, and remained close to control thereafter (Fig. 5).

**Figure 6:**
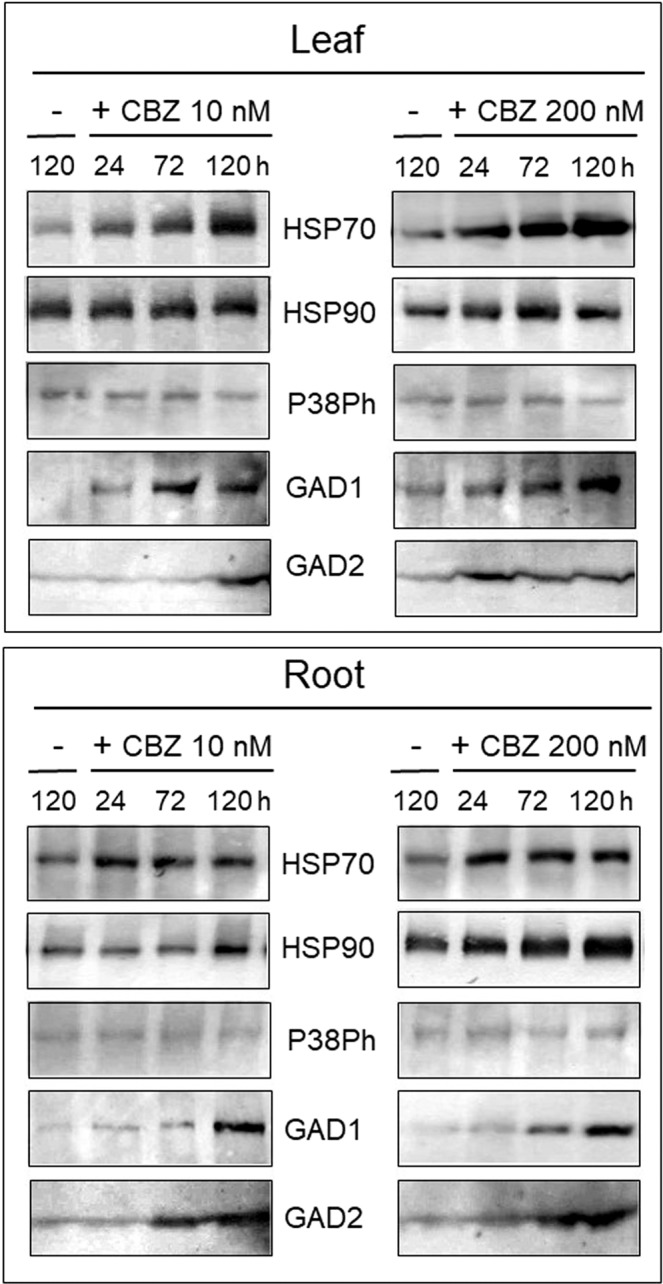
Western blot analyses of tomato leaf and root stress-related protein profiles upon treatment with 10 and 200 nM Carbamazepine (CBZ) for up to 120 h. Roots of tomato seedlings were bathing in tap water (−) and in CBZ (+) at 10 and 200 nM, as indicated. Total proteins were extracted from leaves (upper panel) and roots (lower panel) harvested after 24, 72, and 120 h (roots bathing in tap water for 120 h served as control). Each set of samples was subjected to electrophoresis in five identical gels. Each gel was blotted and reacted with a different stress-related protein antibody raised against chaperones (HSP70, HSP90), the member of the MAPK family phosphorylated P38 (P38Ph), and of the GABA-shunt enzymes glutamate decarboxylases 1 and 2 (GAD1 and GAD2). The photographs show the relevant region of the gel.

The amounts of the GABA-shunt enzymes, GAD1 and GAD2 remained quasi unchanged during 24 h in the presence of 200 or 1000 nM CBZ (shown for 200 nM in Fig. 5). Only the exposure of roots for 24 h to 4000 nM CBZ led to an increase of GAD2 amounts, but not of GAD1 (Supplemental Fig. 1b). The effects of long-term exposure to CBZ were analyzed in leaf and root tissues of seedlings incubated during 24, 72 and 120 h in 10 and 200 nM of CBZ (Fig. 6). Proteins extracted from plants hold in water for 120 h were used as control in immunodetection studies. CBZ concentrations of 10 and 200 nM led to prominent accumulation of HSP70 in leaf and root HSP90 remained at the same levels in leaves, but accumulated in roots. Phosphorylation of MAPK P38 was not detected under CBZ-induced stress, while GAD1 and GAD2 noticeably accumulated in both tissues.

#### Amino acids patterns

**Figure 7:**
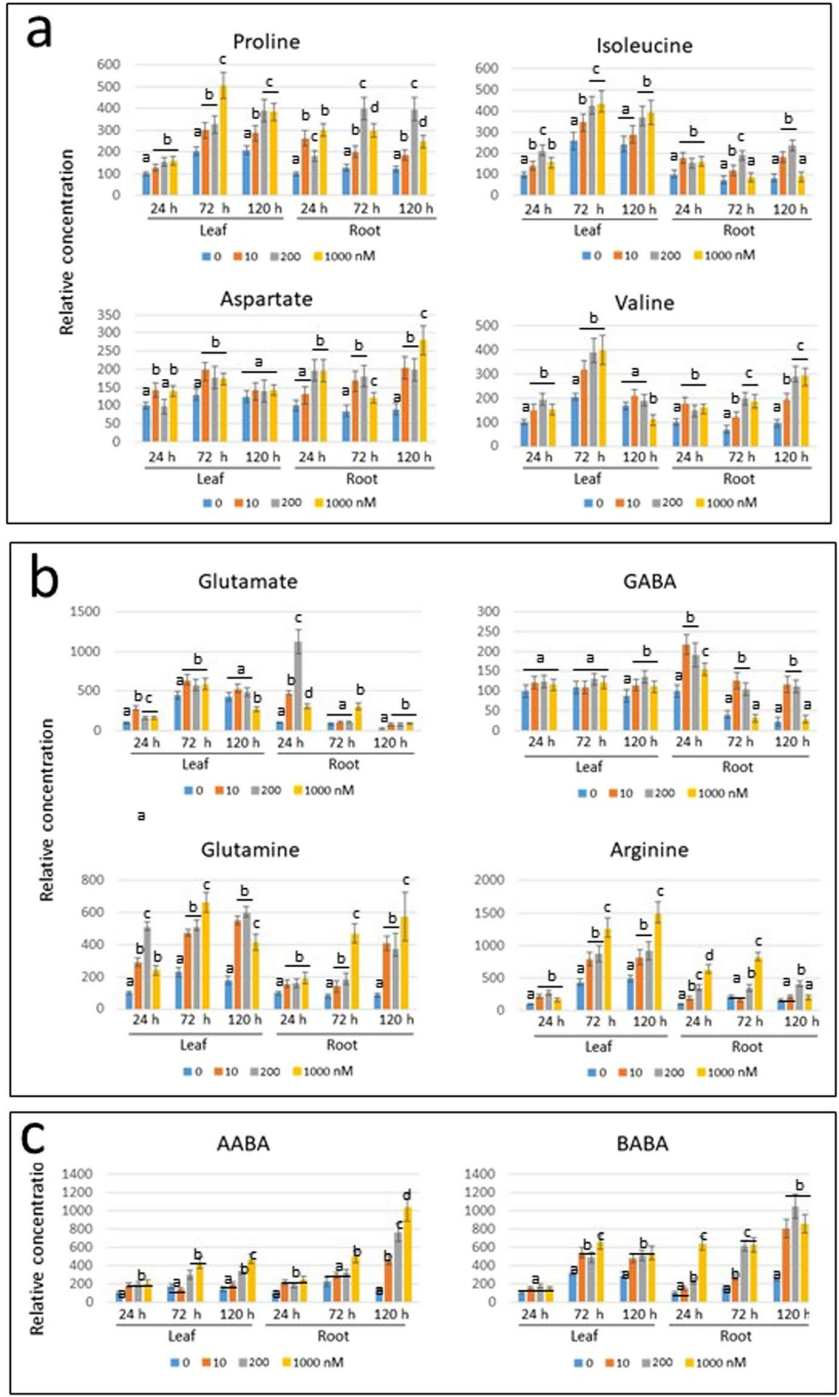
Amino acid profile of tomato leaf and roots with roots bathing in 0, (tap water) 10, 200 and 1000 nM of CBZ. The leaves and roots were sampled after 24, 72 and 120 h. The concentration of amino acids in leaf and root of plants incubated in water was considered as 100; the concentrations of amino acids were calculated relative to this value. **(a)** Osmo-protectants, which include Pro, Ile, Asp, Val. **(b)** Glutamate family of amino acids, which includes Glu, Gln, GABA and Arg, common osmolytes accumulated by plant cells in response to stress. **(c)** Non-protein amino acids α- and β-aminobutaric acids (AABA and BABA, respectively) involved in pathogen resistance. Three independent experiments were performed. The amounts at every time-point/pollutant concentration were measured three times. Bars represent the standard errors, and different lowercase letters (**a**–**d**) above the bars denote significant differences (p < 0.05).

The influence of increasing concentration of CBZ, 0, 10, 200 and 1000 nM, on the osmo-protectants Pro, Asp, Ile, and Val was studied during 1, 3 and 5 days of treatment (Fig. 7). CBZ appeared to be a very effective inducer of osmolyte amino acids, in leaf as well as in root. An increased content of Pro together with Val and Ile were detected in leaves and roots at all the concentrations of CBZ tested, from 24 to 120 h long treatments. The stimulation of Asp was less apparent in leaf, but unambiguous in root.

The effect of the CBZ concentrations on the Glutamate-family members was appraised (Fig. 7b). Glutamate mildly reacted to CBZ, except by an increase in roots observed at 24 h of incubation. GABA, the derivative of Glu, showed a stable pattern in leaves during the five days of treatment; only in roots did CBZ-induced an increase in GABA levels. In response to CBZ, the amounts of two other Glu derivatives, Gln and Arg, increased in leaf and root. CBZ induced an increase in AABA and BABA, both known to be stress-protectors in plants (Fig. 7c). No remarkable changes in contents of Lys, Thr, Leu and Cys were observed in leaf and root in CBZ-treated tomato seedlings (Supplemental Fig. 2b).

## Discussion

As proposed before^25^, we have postulated that the neuroactive compounds remaining in treated wastewater induce stress in irrigated plants. To test this hypothesis, we have watered tomato seedlings with these pollutants and we have confronted our results with a number of criteria characterizing a general stress response in plants. We have used five cocktails of five pharmaceutical compounds (CBZ, LTG, VAL, PHY, DZP); in each cocktail, the pharmaceuticals were each at a concentration of 0, 10, 100 and 1000 μg/l (0, 10, 100 and 1000 ppb). We have also used CBZ alone at concentrations of 0, 2.3, 47.1 and 235.8 μg/l (0, 10, 200 and 1000 mM). Changes in molecular stress markers such as HSPs, MAPKs, GABA shunt enzymes, GABA and amino acids, were conspicuous in leaf and root of tomato seedlings treated with a cocktail of pharmaceutical compounds (CBZ, LTG, VAL, PHY, DZP) and with CBZ alone. In an experiment conceptually similar to ours^24^, cucumber seedlings in a hydroponic setting were exposed to a mixture of 17 pollutants (including DZP and CBZ), at concentrations of 0, 0.5, 5 and 50 μg/l. At 0.5 and 5 μg/l, ROS levels in roots and shoots increased several folds. At 5 and 50 μg/l, an increase in the activity of antioxidant enzymes such as peroxidase and glutathione-S-transferase was observed.

Stress induces the denaturation of cell proteins. Activation of HSPs protects cells from protein collapse (reviewed in^29^). In plants, HSPs are grouped into classes according to their approximate molecular weight: (a) Hsp100, (b) Hsp90, (c) Hsp70, (d) Hsp60 and (e) small heat-shock proteins (sHsps). The importance of HSPs in establishing tolerance to different stresses was shown in tomato plants^30^. MAPKs are involved in plant response to biotic and abiotic stresses (reviewed in^31^). The extracellular stress signals are transmitted within the cell to the nucleus, via a phosphorylation cascade, which includes MAPK, MAPK kinase (MAPKK) and MAPKK kinase (MAPKKK), activating stress-response genes. P38 is a class of MAPK upregulated in response to different types of cellular stresses.

In tomato plants subjected to the pharmaceutical cocktail for up to 24 h, the amounts of HSP70, HSP90 and MAPK P38Ph increased in leaves (Fig. 2); for longer time, HSP70 amounts increased in leaves, HSP90 - in roots, GAD1 - in leaves and roots, GAD2 - in leaves with a weak increase in roots. The effect of the cocktail on these protein stress markers was dose-dependent; the concentration of 100 ppb caused the most pronounced accumulation (Fig. 3).

In plants treated for 2 to 4 h with CBZ alone (200 nM), an increase in the amounts of HSP70, HSP90 and P38P, but not of GABA-shunt enzymes, was observed in leaf (Fig. 5). The short-term response of these stress markers to CBZ alone and to the drug cocktail were similar. CBZ, even at low concentration (10 nM), reinforced the accumulation of HSP70, HSP90 and GAD2 compared to the cocktail treatment in root (Figs. 5 and 6). Such increase was unexpected. However, pharmaceuticals present only in concentrations that do not provoke significant toxic effects if acting singly can have significant effects on an organism when mixed^32^. This observation holds for very low concentrations of pharmaceutics present in the environment (ng/g dry soil, μg-ng/l in surface water and wastewater treatment plants effluents). In the current study, the lowest cocktail concentration was 10 ppb (10 μg/l); the lowest CBZ concentration was 10 nM (2.358 μg/l), comparable to the concentration of 1.3 to 2.2 μg/l recently measured in treated wastewater used to irrigate tomatoes in Israel^19^. Therefore, it seems that, at such concentrations, the toxicity of a single component is as potent as a drug mixture. The other parameters are time of treatment, which in real field conditions is much more prolonged than the five days experimented in the study, and the presence of other environmental stresses such as heat.

Stress response in plants also involves changes in the metabolism of protein and non-protein amino acids^33^. Abiotic stress tolerance can be achieved by proline (Pro) synthesis to counter osmotic stress^34^, making Pro a stress indicator. Besides Pro and its derivatives, glycine betaine (GB), valine (Val), isoleucine (Ile) and aspartic acid (Asp) are major organic osmolytes that accumulate in a variety of plant species in response to environmental stresses such as drought, salinity, extreme temperatures, UV radiation and heavy metals^35^. A meta-study on the metabolic response of *Arabidopsis* to abiotic stresses revealed that lysine (Lys) and threonine (Thr) are also induced under several stress conditions^36^. Recent research on Durum wheat subjected to high salinity demonstrated the central role of GABA in addition to Pro and GB as a major osmolyte^37^. Moreover, the synthesis of GABA and of other amino acids, including Pro, remodeled the metabolism and the defense processes, playing a key role in the response to simultaneous stresses^38^.

The tomato seedlings subjected to pharmaceutical treatments showed a typical stress-induced pattern of those amino acids known to be plant stress markers (Figs. 4 and 7). The response was differential in its strength, time, and tissue specificity. The main osmolyte Pro was highly induced in roots, but not in leaves. CBZ was able to induce Pro accumulation even after 120 h, while its induction by the cocktail treatment was minor. Other osmoprotectants, such Val, Ile and Asp, were more effectively induced by CBZ in roots than by the cocktail.

Similar to the pharmaceutical cocktail, CBZ led to minor changes in Glu (only after 24 h). However, Gln amounts were increased in roots following treatment by the cocktail and by CBZ. CBZ induced Arg accumulation. There was no essential CBZ-dependent increase of GABA concentration in leaves, while the cocktail of pharmaceuticals caused GABA induction after 72 and 120 h (Figs. 4 and 7). In roots, a pronounced increase of GABA occurred after both treatments. In response to CBZ, AABA and BABA accumulated, which suggested the potential of acquired resistance against different tomato pathogens^39^.

The isomers of aminobutyric acid (AABA, BABA, GABA) accumulate at different levels in plants as a response to various stresses^40^. GABA activates the defense system to cope with abiotic (heat, drought, salt) and biotic (virus, bacteria, fungi, insects) stresses. AABA and BABA induce PR proteins^41–43^, while BABA induces ROS and stops pathogen colonization^39^.

It was shown previously in tomato plants that GABA, Asp, Glu and Gln accumulate in response to stresses^44^. Glu plays a central role in plant amino acid metabolism, providing both the C skeleton and the α-amino group for the biosynthesis of amino acids with key roles in plant defense, such as GABA, Arg, and Pro^44^. GABA eases the effects of stress through various mechanisms^45^. Extreme temperatures and drought induce the acidification of the cytosol, provoking GAD activation and GABA synthesis. Plants, like animals, may possess GABA-like receptors. Animal GABA receptors may serve as model to understand the stress-related ion concentration changes that leads to GABA induction and the role of GABA as messenger from stress to stress response^46^. In the current study, the effects of psychoactive drugs, especially CBZ, on concentrations of GABA and GABA-shunt enzymes, GAD1 and GAD2, pointed to possible interactions of CBZ with plant GABA receptors.

There is an increasing body of evidence showing that components of the GABA shunt protects plants against simultaneous environmental stresses^47^. In humans, the GABA-shunt is involved in specific cellular responses to pharmaceuticals. In mammals, psychoactive drugs such as DZP enhance the effect of the GABA neurotransmitter^48,49^. DZP is a positive allosteric modulator of the GABA type A receptors (GABAA). The drug enhances the response to GABA by opening GABA-activated chloride channels and allowing chloride ions to enter the neuron, making the neuron negatively charged and resistant to excitation^48,49^.

The GABA shunt genes and amino acids were investigated in rice^50^ and in tomato^51^. We used antibodies raised against rice GADs to investigate tomato GADs. Despite the high amino acid homologies among GADs, and between GADs from tomato and rice, we have confirmed the specificity of tomato GAD1 and GAD2 by quantitative RT real-time PCR (qPCR). For example, PCR analyses of *SlGAD1* and *SlGAD2* gene transcripts of leaves from untreated (0 nM) and CBZ-treated (10 nM) tomato plants paralleled the protein patterns seen in Fig. 6 (Supplemental Fig. 3).

Altogether, the changes in the patterns of the main stress response markers caused by pharmaceutical treatment, especially with CBZ, allowed us to conclude that these compounds are to some extent toxic for tomatoes. Plant cells mobilize the protection machinery to avoid changes in their metabolism and to restore homeostasis^52^. Accumulation of stress markers during prolonged treatments points to a growing stress response with consequent changes in cellular metabolism. As a potential defense, drugs treatment may lead to protein degradation. Degradation of highly abundant proteins such as subunits of photosystems and ribosomes contributed to an accumulation of free amino acids. However, the patterns of ubiquitinated proteins (UPS) and ATG8 (autophagy) were not significantly changed in tomato cells under mild pharmaceutical stress (mix 10–100 ppb, CBZ 10–200 nM) during 1–5 days (not shown).

The risk assessment for tomatoes, and for other plants, irrigated with treated wastewater containing these pharmaceuticals, seems to depend on the pharmaceutical concentrations and on the duration of irrigation. The current study was limited to five days of treatments, because the main goal was to uncover the stress response. Prolonged incubations with pharmaceutical compounds are required to appraise their long-term effects, for example, under hydroponic growth conditions, which allows maintaining plants for up to 50–60 days. The concentrations of pharmaceuticals in this study were similar or higher than those measured in treated wastewater used for irrigation in Israel (about 2 μg CBZ/l^21^. However, there may be much more than five pharmaceuticals in wastewater used for field irrigation. The toxic potential of a compounds depends not only on concentration of the single drug, but also on properties of diversity of pharmaceuticals in wastewater and irrigated soil, sorption capacity and kinetics, pH of soil, biodegradation rate of compounds, and the presence of other elements in water and soil^25^.

The other risk concerns the alteration of plant tolerance to environmental stresses. The increased amounts of osmolytes could lead to higher plant resistance to drought^53^. On the other hand, increased amounts of GABA could lead to sensitivity to some insect pathogens^54^, while accumulation of BABA may induce tolerance to some biotic and abiotic stresses^39,44,45^.

## Materials and Methods

### Plants

Tomato (*Solanum lycopersicum*, cv. Daniella) plantlets, 3 weeks after sowing, were used.

### Pharmaceuticals treatment

Three-weeks old tomato seedlings were removed from the nursery tray and the roots were gently washed with running water. The roots were left to bath in a 50 ml Falcon tube containing a mixture of five pharmaceutical compounds: Carbamazepine (CBZ), Lamotrigine (LTG), Valproic acid (VAL), Phenytoin (PHY), and Diazepam (DZP). Mixtures at three concentrations were used; in each, the five compounds were present at the same concentration: 10, 100, and 1000 ppb (1 ppb is 1 μg/l). Tomatoes were also treated the same way with CBZ alone, at three concentrations: 10, 200 and 1000 nM (1000 nM is 235.8 μg/l). Control plants had roots bathing in tap water originating from the Coastal Plain aquifer. It had a of pH 7.0 to 7.4, an EC of 1.0 to 1.4 dS/m and was non-fluoridated. We did not add minerals or fertilizers. The solutions were replaced every 2 days. Each treatment with the mixture of pharmaceuticals and with CBZ alone was repeated at least 5 times. For each experimental point, three Falcon tubes, each containing one seedling, were used. Leaf and root samples (about 0.5 g each) were composed of mixes of tissue pieces from the treated plants. Samples of leaves and roots were immediately frozen in liquid nitrogen and stored at −80 °C.

### Immunodetection of stress-related proteins

Western blotting and immunodetections were performed as described before^55^. About 50 mg of frozen leaves and roots (pooled from three plantlets per Falcon tube, see above) were minced and drill-homogenized in a standard SDS-PAGE loading buffer supplemented with 2% SDS. Samples were boiled for 10 min and centrifuged for 10 min at 10,000 × *g*; about 30 μg protein from the supernatants were subjected to SDS-polyacrylamide gel electrophoresis (SDS-PAGE). Antibodies against HSP70, HSP90, HSP100/ClpB were purchased from Agrisera (Sweden). Anti-GAD1 and GAD2 polyclonal antibodies were prepared against expressed rice (Oryza sativa) recombinant proteins from pET32a::full-length cDNA of OsGAD1 or OsGAD2 (Accession no.: AB056060 (GAD1) and AB056061 (GAD2)^56^. Incubation with primary antibodies was followed by exposure to secondary goat peroxidase coupled antibodies (Agrisera, Sweden) and by ECL detection (Amersham, UK). Each immunodetection was repeated at least three times for each set of plants. Blots were documented using an ImageQuant LAS500 imager (GE Healthcare Life Sciences). Only the relevant part of the blot is shown in the figures. In a given figure, results are from a single run and were not cropped from two or more gels. Images are framed to indicate that each gel is independent from the others.

### Amino acid quantification

Fifty mg of tomato root and leaf were used for each replicate. Three independent experiments were performed. For each time/concentration point, analyzes were done in triplicates. Amino acids were quantified according to the procedure described previously^57^. Acetonitrile and formic acid of ULC/MS grade were from Bio-Lab (Israel). Water with resistivity 18.2 MΩ was obtained using Direct 3-Q UV system (Millipore). Mix of amino acids standards from Sigma-Aldrich was used. Derivatization with AccQTag Ultra kit was performed by following the procedure prescribed by the company (Waters (TC) Israel Ltd.). Namely, 20 μl of AccQTag reagent was added to 10 μl of sample in 70 μl borate buffer, and the mixture was stirred at 55 °C for 10 min. Before LCMC analysis the samples were filtered through 0.2-μm PVDF-filters (Millex GV) to rid of insoluble material. The LC-MS/MS instrument consisted of Acquity I-class UPLC system (Waters) and Xevo TQ-S triple quadrupole mass spectrometer (Waters) equipped with an electrospray ion source and operated in positive ion mode was used for analysis. MassLynx and TargetLynx software (v.4.1, Waters) were applied for the acquisition and analysis of data. Chromatographic separation was done on a 100 × 2.1-mm i.d. 1.8-µm UPLC HSS T3 column equipped with 50 × 2.1-mm i.d., 1.8-µm UPLC HSS T3 pre-column (both Waters Acquity) with 0.1% formic acid as mobile phase A and 0.1% formic acid in acetonitrile as B at a flow rate of 0.6 ml/min and column temperature 45 °C. A gradient was as follows: 0.5 min the column was hold at 4% B, then linear increase to 10% B in 2 min, then to 28% B in 2.5 min, and to 95% B in 0.1 min. Just after back to 0% B during 1.1 min, and equilibration at 4% B for 1.3 min. Samples kept at ambient temperature (23 °C) were automatically injected in a volume of 1 μl. For mass spectrometry argon was used as the collision gas with flow 0.10 ml/min. The capillary voltage was set to 3.00 kV, cone voltage 25 V, source offset 30 V, source temperature 150 °C, desolvation temperature 650 °C, desolvation gas flow 800 L/hr, cone gas flow 150 L/hr. Analytes were detected using corresponding selected reaction monitoring (SRM) and retention times. The concentrations based on standard curves were calculated using TargetLynx (Waters).

### Statistical analyses

Statistical analysis of amino acid amounts (Figs. 4 and 7, and Supplemental Fig. 2) were as follows. Three independent biological replicates were used in each experiment, and the amino acid amounts were determined as three technical replicates. Comparisons were performed between control and the different concentrations of pharmaceuticals with each time point. Each tissue and time point was considered as an independent experiment, therefore a one-way analysis of variance using drug concentration as factor was conducted using the statistical analysis software package JMP Pro Version 10.0 (SAS Inc., NC, USA, https://www.jmp.com/en_us/software/data-analysis-software.html). Error bars represent standard deviation obtained with Excel spreadsheet.

## Supplementary information

Supplementary information.
