# Supplementary material for: Pharmaceuticals in treated wastewater induce a stress response in tomato plants
Source: Sci Rep. 2020 Feb 5;10:1856. doi: 10.1038/s41598-020-58776-z (PMC7002738; doi:10.1038/s41598-020-58776-z)
Supplement: Supplementary file 1 — Supplementary information. [file 41598_2020_58776_MOESM1_ESM.pdf]

Supplementary information

Pharmaceuticals in treated wastewater induce a stress response in tomato plants

Rena Gorovits [1], Iris Sobol [1], Kazuhito Akama [2], Benny Chefetz [3], Henryk Czosnek [1 a]

[1] Institute of Plant Sciences and Genetics in Agriculture, Robert H. Smith Faculty of Agriculture, Food and Environment, The Hebrew University of Jerusalem, Rehovot 76100, Israel.
[2] Department of Biological Science, Shimane University, Matsue, Shimane 690-8504, Japan
[3] Institute of Soil and Water sciences, Robert H. Smith Faculty of Agriculture, Food and Environment, The Hebrew University of Jerusalem, Rehovot 76100, Israel.
[a] Corresponding author hanokh.czosnek@mail.huji.ac.il

Supplemental Figure 1. Western blot analyses of tomato leaf stress-related protein profiles upon treatment with the pharmaceutical cocktail. (a) Immunodetection of P38Ph in leaves of tomato bathing in tap water (-) and 100 ppb cocktail (+) after 73, 74,76 and 80 h of treatments in two independent experiments (I and II). **(b). Upper panel:** Immunodetection of HSP70 and HSP 90 in tomato leaves with roots bathing for 24 h in tap water used as control (-) and for 1, 2, 4, 8 and 24 CBZ (+). **Lower panel**: GAD2 was similarly immunodetected but with roots bathing in 4000 nM CBZ.

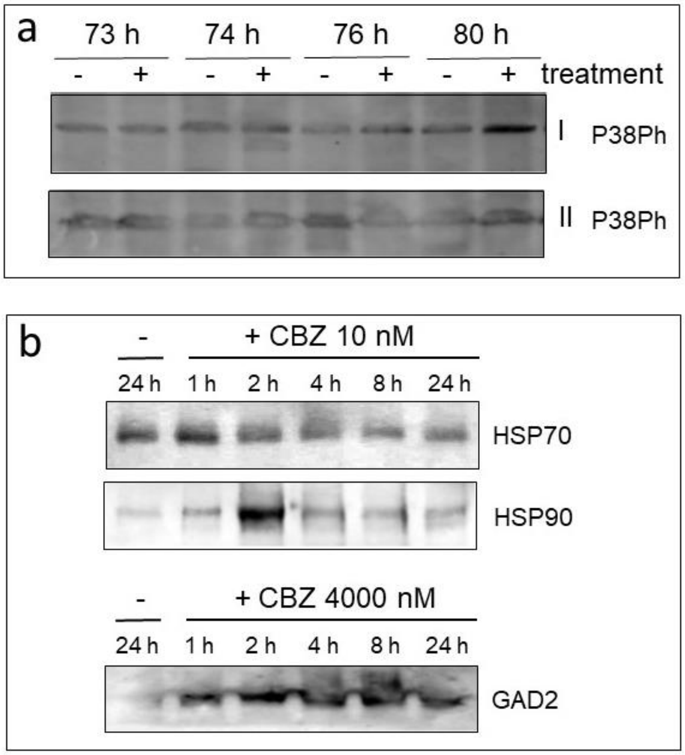
a
73 h
74 h
76 h
80 h
- + - + - + - + treatment
I P38Ph
II P38Ph
b
-
+ CBZ 10 nM
24 h 1 h 2 h 4 h 8 h 24 h
HSP70
HSP90
-
+ CBZ 4000 nM
24 h 1 h 2 h 4 h 8 h 24 h
GAD2

Supplemental Figure 2. Amino acid profile of tomato leaf and roots of tomato bathing in (a) pharmaceutical cocktail at 0 (tap water), 10, 100 and 1000 ppb, and (b) in CBZ at 0, 10, 200 and 1000 nM. The leaves and roots were sampled after 24, 72 and 120 h. The concentration of Lys, Thr, Leu and Cys in leaf and root of plants incubated in water was considered as 100; the concentrations of amino acids were calculated relative to this value. Three independent experiments were performed. The amounts at every time-point/pollutant concentration were measured three times. Bars represent the standard errors, and different lowercase letters (a–d) above the bars denote significant differences ($p<0.05$).

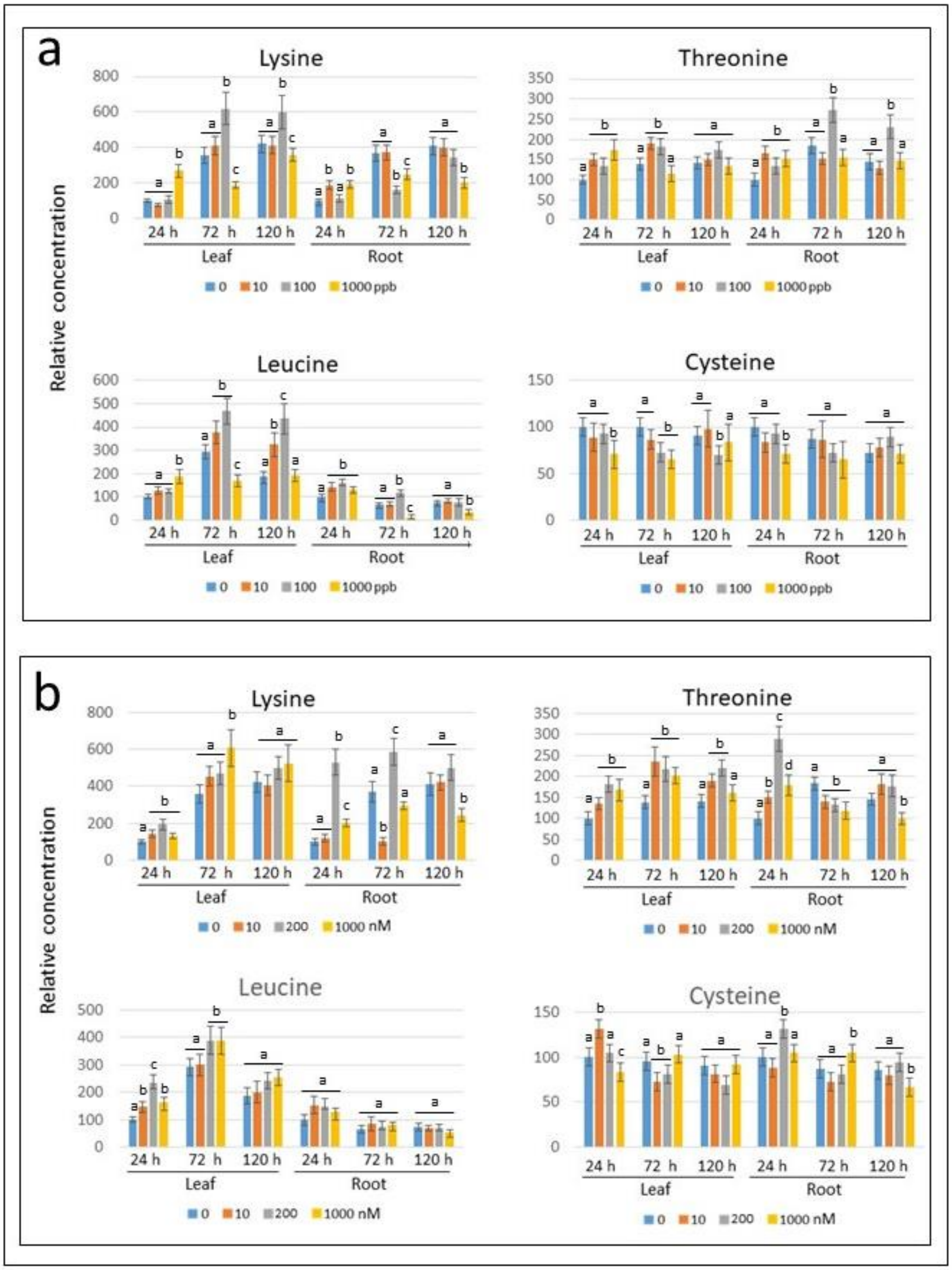
a
Lysine
Threonine
Leucine
Cysteine
Relative concentration
24 h
72 h
120 h
Leaf
Root
0
10
100
1000 ppb
b
Lysine
Threonine
Leucine
Cysteine
Relative concentration
24 h
72 h
120 h
Leaf
Root
0
10
200
1000 nM

Supplemental Figure 3. Expression of tomato *SlGAD1* and *SlGAD2* mRNAs (a) and GAD1 and GAD2 proteins (b) in leaves of tomato plants treated with 10 nM CBZ, and sampled after 0, 72 and 120 h. The RNAs were extracted from the leaf samples Western blot analyzed in Figure 6 using rice GAD1 and GAD2 antibodies (**b**). CBZ 10 nM was chosen because this concentration showed the greatest difference between GAD1 and GAD2. The expression levels of tomato *GAD1* and *GAD2* genes were calculated in relation to untreated leaves, taken as 1. The results were normalized using the tomato *β-actin* gene as an internal marker. Bars represent the average and standard deviation of the relative expression from three independent biological repeats; pooled leaves of three different plants were taken for each sample. The qPCR primer pairs were: SlGAD1-12F: 5' AAACTTCCCATTTCCCAACC 3' and SlGAD1-117R: 5' CGATTGATCGGAGGAGAAAA 3'; SlGAD2-35F: 5' CTTTGATCTTCTCCGTCGTTG 3' and SlGAD2-139R: 5' ATATCGAGACGCGAAAGTCG 3' (51).

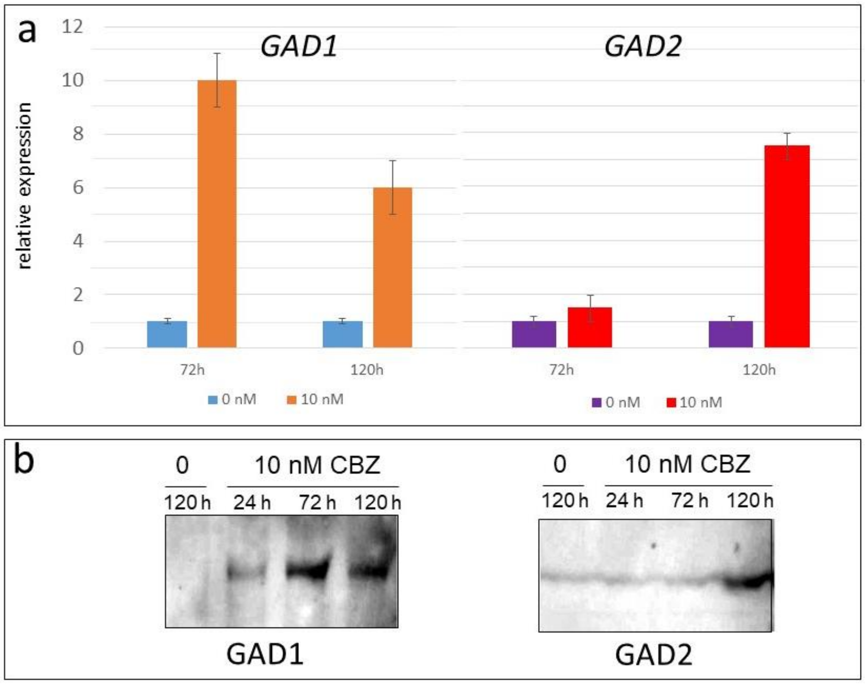
a
GAD1
GAD2
relative expression
12
10
8
6
4
2
0
72h
120h
72h
120h
0 nM
10 nM
0 nM
10 nM
b
0
10 nM CBZ
120 h
24 h
72 h
120 h
GAD1
0
10 nM CBZ
120 h
24 h
72 h
120 h
GAD2
